# Prognostic Value of BUN-Based Ratios for Mortality and Prolonged Hospitalization in Acute Upper Gastrointestinal Bleeding: Comparison with Established Risk Scores

**DOI:** 10.3390/medicina62061210

**Published:** 2026-06-22

**Authors:** Bayram İnan, Çağdaş Erdoğan, Emir Tuğrul Keskin, Yavuz Özden, Hulusi Can Karpuzcu, İhsan Ateş, Zeki Mesut Yalın Kılıç

**Affiliations:** 1 Department of Gastroenterology, Ankara Bilkent City Hospital, Ankara 06800, Türkiye; emirkeskin.md@gmail.com (E.T.K.); zmykilic@yahoo.com (Z.M.Y.K.); 2 Department of Gastroenterology, School of Medicine, Istinye University, Istanbul 34093, Türkiye; cagdas_erdogan@hotmail.com; 3 Department of Gastroenterology, Kayseri City Hospital, Kayseri 38080, Türkiye; yavuzozden@gmail.com; 4 Department of Gastroenterology, Ankara Etlik City Hospital, Ankara 06800, Türkiye; can.karpuzcu@gmail.com; 5 Department of Internal Medicine, Ankara Bilkent City Hospital, Ankara 06800, Türkiye; dr.ihsanates@hotmail.com

**Keywords:** acute upper gastrointestinal bleeding, BUN/Hb, BUN/albumin, mortality, prolonged hospitalization, AIMS-65, Glasgow–Blatchford score, ABC score, Rockall score

## Abstract

*Background and Objectives*: This study investigated the prognostic value of two simple blood urea nitrogen (BUN)-based ratios, BUN/hemoglobin (Hb) and BUN/Albumin, for predicting in-hospital mortality and prolonged hospitalization in patients with acute upper gastrointestinal bleeding (UGIB). Their performance was compared with established risk scores, including the Glasgow–Blatchford score (GBS), AIMS-65, ABC and Rockall scores. *Materials and Methods*: This retrospective cohort study included 486 patients evaluated for acute UGIB between March 2023 and February 2026. The diagnostic performance of BUN/Hb and BUN/Albumin ratios was assessed using receiver operating characteristic (ROC) analysis and compared with established risk scores. Associations with clinical outcomes were evaluated using logistic regression analyses. *Results*: The median age was 67 years, and 292 patients (60.1%) were male. In-hospital mortality occurred in 17 patients (3.5%), while prolonged hospitalization was observed in 207 patients (42.6%). AIMS-65 showed the highest Area Under the Curve (AUC) for mortality prediction (0.799; 95% CI 0.696–0.902), followed by the ABC score (0.731) and the BUN/Albumin ratio (0.711). For prolonged hospitalization, BUN/Hb showed the highest AUC (0.706; 95% CI 0.660–0.752), although differences from established scores were not statistically significant. In multivariable analysis, BUN/Albumin remained associated with mortality, whereas BUN/Hb did not reach statistical significance for prolonged hospitalization. However, mortality-related findings should be interpreted with caution because only 17 in-hospital deaths occurred in the study cohort. *Conclusions*: Simple BUN-based ratios may provide complementary prognostic information in acute UGIB. BUN/Albumin was associated with in-hospital mortality and showed modest discriminatory ability, but it did not demonstrate statistically significant superiority over established risk scores. BUN/Hb showed the numerically best discrimination for prolonged hospitalization, but without statistically significant superiority or persistent significance in multivariable analysis. Overall, these ratios may serve as supportive tools for early risk assessment rather than replacements for established risk scoring systems.

## 1. Introduction

Acute upper gastrointestinal bleeding remains a clinically important, serious emergency condition that requires rapid decision-making [1]. Patients may present with various clinical manifestations, including melena, hematemesis, hematochezia, syncope, or hemodynamic instability [2]. While some patients become clinically stable after short-term observation and supportive treatment, others may require blood transfusion, endoscopic intervention, intensive care monitoring, or prolonged hospitalization. Therefore, estimating the patient’s risk level at presentation is of great importance both for predicting the clinical course and for ensuring the efficient use of healthcare resources [3].

Several scoring systems are available for risk assessment in upper gastrointestinal bleeding. The GBS, AIMS-65, ABC score, and Rockall score are among the most commonly used tools in this setting. These scores assist clinicians in estimating mortality, rebleeding risk, transfusion requirement, need for endoscopic intervention, and hospitalization decisions. However, each of these systems demonstrates varying degrees of performance in predicting different clinical outcomes [4,5,6,7]. For example, some scores are more useful for identifying low-risk patients, whereas others may better reflect mortality risk. This suggests that a single scoring system may be insufficient to address all clinical needs in acute upper gastrointestinal bleeding. In daily clinical practice, in addition to risk scores, simple, readily available, and rapidly interpretable laboratory parameters play an important role in clinical decision-making. In this context, blood urea nitrogen, hemoglobin, and albumin levels are of particular interest. BUN levels in gastrointestinal bleeding may reflect both the absorption of digested blood proteins and renal perfusion and circulatory status [8]. While hemoglobin provides essential information regarding the degree of blood loss, albumin is associated with the patient’s nutritional status, inflammatory burden, and overall physiological reserve [9]. Therefore, combining BUN with hemoglobin or albumin as a ratio may provide a more comprehensive understanding of the patient’s clinical condition than single measurements alone. Nevertheless, the prognostic value of BUN-based ratios in acute upper gastrointestinal bleeding has not yet been defined as clearly as that of established risk scores. Although current scoring systems are more comprehensive, some require multiple clinical variables or endoscopic findings. In contrast, the BUN/Hb and BUN/Albumin ratios can be easily calculated from routine laboratory tests and may guide clinicians in the early phase of patient assessment. Particularly in busy emergency department settings, whether such rapidly accessible biochemical ratios can contribute to risk stratification is clinically important [8,10]. Although mortality is one of the most frequently evaluated outcomes in studies of upper gastrointestinal bleeding, prolonged hospitalization is also a clinically relevant outcome that deserves attention [11]. Prolonged hospitalization reflects not only the severity of bleeding, but also the patient’s comorbidity burden, response to treatment, inflammatory status, need for transfusion and intervention, and utilization of hospital resources [11,12]. Therefore, identifying simple markers that may predict length of hospital stay could contribute to the planning of clinical management.

In this study, we investigated the prognostic value of two simple BUN-based ratios, namely the BUN/Hb and BUN/Albumin ratios, in predicting in-hospital mortality and prolonged hospitalization among patients evaluated for acute upper gastrointestinal bleeding. In addition, the performance of these ratios was compared with established risk scores, including GBS, AIMS-65, ABC, and Rockall. Thus, the aim of the study was to determine whether these ratios, which can be easily derived from routine laboratory data, provide additional prognostic value to current risk stratification approaches.

## 2. Materials and Methods

This study was designed as a retrospective cohort study. Patients diagnosed with upper gastrointestinal bleeding based on clinical, laboratory, and endoscopic findings at the Gastroenterology Clinic of Ankara Bilkent City Hospital between March 2023 and February 2026 were included. Patients aged 18 years or older with a diagnosis of upper gastrointestinal bleeding were eligible for inclusion. The exclusion criteria were as follows: (1) incomplete clinical, laboratory, or endoscopic data preventing definitive diagnosis; (2) pregnancy; and (3) lower gastrointestinal bleeding.

After applying the inclusion and exclusion criteria, 486 patients were included in the study. The study was conducted in accordance with the Declaration of Helsinki and was approved by the Ethics Committee of Ankara Bilkent City Hospital (TABED 1-26-2413).

### 2.1. Data Collection

All data were retrospectively obtained from the hospital electronic medical records. Demographic variables, including age and sex, were recorded for each patient. Comorbidities included hypertension, diabetes mellitus, coronary artery disease, atrial fibrillation, cerebrovascular disease, and dementia. Clinical presentation findings, including melena, hematemesis, hematochezia, and syncope, were recorded for each patient. Medication use, including antihypertensive agents, antiplatelet agents, anticoagulants, oral antidiabetic drugs, statins, and insulin, was also documented.

Vital signs, including systolic and diastolic blood pressure and heart rate, were recorded. Laboratory parameters included admission hemoglobin level, white blood cell count, platelet count, red cell distribution width, neutrophil count, lymphocyte count, and monocyte count. Based on these parameters, the neutrophil-to-lymphocyte ratio, platelet-to-lymphocyte ratio, lymphocyte-to-monocyte ratio, and monocyte-to-lymphocyte ratio were calculated. Other laboratory variables, including BUN, creatinine, albumin, INR, CRP, aspartate aminotransferase, alanine aminotransferase, total bilirubin, direct bilirubin, alkaline phosphatase, lactate dehydrogenase, and gamma-glutamyl transferase, were also recorded. Serum urea values reported by the hospital laboratory were converted to blood urea nitrogen values using the standard formula BUN (mg/dL) = serum urea (mg/dL) × 0.467, and the converted BUN values were used in all subsequent ratio calculations.

Clinical risk scores, including the GBS, AIMS-65, ABC score, and Rockall score, were calculated [4,5,6,7]. The Rockall score used in the present analysis was the full Rockall score, incorporating both clinical and endoscopic components. In addition, the BUN/Hb and BUN/Albumin ratios were calculated as laboratory-based comparative markers. Endoscopic findings were evaluated and recorded.

Regarding clinical outcomes, length of hospital stay, in-hospital mortality, and prolonged hospitalization were assessed. Prolonged hospitalization was defined as a length of hospital stay of ≥7 days, based on previous UGIB studies using a 7-day threshold for prolonged hospital stay [11,13]. The diagnostic performance of BUN/Hb, BUN/Albumin, and established risk scores, including the GBS, AIMS-65, ABC score, and Rockall score, in predicting clinical outcomes was evaluated using ROC analysis. The clinical outcomes of interest were in-hospital mortality and prolonged hospitalization.

### 2.2. Statistical Analysis

Continuous variables are presented as median with interquartile range (IQR), and categorical variables as frequency with percentage. All continuous variables were assessed for normality using the Shapiro–Wilk test; none met normality criteria, and non-parametric descriptive measures were used throughout.

The BUN/Hb ratio was computed as BUN (mg/dL) divided by admission hemoglobin (g/dL), and the BUN/Albumin ratio as BUN (mg/dL) divided by serum albumin (g/dL). The discriminative ability of each BUN-Based Ratios and each established scoring system for in-hospital mortality and prolonged hospitalization (≥7 days) was assessed using ROC analysis. AUC values with 95% confidence intervals were calculated by the DeLong method. Optimal cutoff values were determined by the Youden index (J = sensitivity + specificity − 1), and corresponding sensitivity, specificity, positive predictive value (PPV), and negative predictive value (NPV) are reported. Pairwise AUC comparisons between BUN-Based Ratios and each established score were performed using the DeLong test.

Univariate logistic regression was performed for all candidate clinical, laboratory, and scoring variables. Continuous predictors are reported as odds ratios (OR) per clinically meaningful unit increments. Variables significant at *p* < 0.05 in univariate analysis were considered candidates for multivariate modeling. Final multivariate models were constructed using backward stepwise selection with a retention threshold of *p* < 0.05. To reduce the risk of overfitting, particularly in the mortality analysis with only 17 events, the number of predictors included in the final multivariable models was deliberately restricted. Candidate variables were selected according to univariate significance and clinical relevance. Collinearity among BUN, hemoglobin, albumin, BUN/Hb, BUN/Albumin, and established risk scores was assessed using correlation analysis and variance inflation factors. Variables with direct mathematical or substantial conceptual overlap were not entered simultaneously into the same multivariable model. To assess the potential added value of BUN-Based Ratios, a base model (Model A) comprising established clinical predictors was compared with an extended model (Model B) incorporating the corresponding BUN-based ratio. This study primarily compared the discriminative performance of BUN-based ratios with established risk scores using ROC analysis. Formal incremental value analyses, such as adding BUN/Hb or BUN/Albumin to validated risk scores and assessing model improvement, were not performed and should be addressed in future studies.

Given the limited event count for mortality (*n* = 17), the number of predictors in each model was deliberately restricted to reduce the risk of overfitting. Model goodness-of-fit was assessed using the Hosmer–Lemeshow test; a *p*-value greater than 0.05 was considered indicative of adequate calibration.

All statistical analyses were performed using Python software (version 3.11.1 Python Software Foundation, Wilmington, DE, USA) with the scikit-learn and SciPy libraries. A two-tailed *p*-value of less than 0.05 was considered statistically significant.

## 3. Results

### 3.1. Study Population and Baseline Characteristics

A total of 486 patients who met the inclusion criteria and had complete clinical, laboratory, and endoscopic data were included in the final analysis. Baseline characteristics are summarized in Table 1. The median age was 67 years (IQR 49–79), and 292 patients (60.1%) were male. Comorbid conditions were common, including hypertension in 238 patients (49.0%), coronary artery disease in 149 (30.7%), and diabetes mellitus in 133 (27.4%). Antiplatelet and anticoagulant agents were used by 158 (32.5%) and 111 (22.8%) patients, respectively.

Melena was the predominant presenting symptom (72.6%), followed by hematemesis (40.3%). Median admission hemoglobin was 8.80 g/dL (IQR 7.20–11.00) and median BUN was 13.27 mg/dL (IQR 8.97–20.02). Endoscopy revealed peptic ulcer disease in 247 patients (50.8%), with high-risk stigmata (Forrest 1A–2B) identified in 93 (19.1%). Gastritis accounted for 38.9% of endoscopic diagnoses. Median GBS, AIMS-65, ABC, and Rockall scores were 7, 1, 2, and 4, respectively. The BUN/Hb ratio had a median of 1.62 (IQR 0.92–2.54) and the BUN/Albumin ratio had a median of 3.68 (IQR 2.29–5.61).

### 3.2. Clinical Outcomes

In-hospital mortality occurred in 17 patients (3.5%). Prolonged hospitalization, defined as a stay of seven or more days, was observed in 207 patients (42.6%). Median length of hospital stay was 5 days (IQR 2–10).

### 3.3. Diagnostic Performance of BUN-Based Ratios and Established Scoring Systems

**Figure 1:**
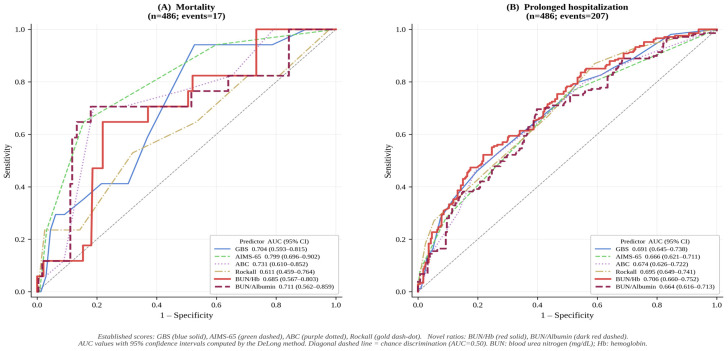
ROC curves for BUN-based ratios and established scoring systems in predicting clinical outcomes in patients with acute upper gastrointestinal bleeding (*n* = 486). Notes: (**A**) In-hospital mortality (events = 17). (**B**) Prolonged hospitalization ≥ 7 days (events = 207). Established scores: GBS (blue solid), AIMS-65 (green dashed), ABC (purple dotted), Rockall (gold dash-dot). BUN-Based Ratios: BUN/Hb (red solid), BUN/Albumin (dark red dashed). AUC values with 95% confidence intervals (DeLong method) are displayed in each panel legend. The diagonal dashed line represents chance-level discrimination (AUC = 0.50). AUC: area under the curve; BUN: blood urea nitrogen (mg/dL); Hb: admission hemoglobin; GBS: Glasgow–Blatchford Score; AIMS-65: Albumin, INR, Mental status, Systolic blood pressure, age ≥ 65; ABC: Acuity of Upper GI Bleeding score.

The AUC values for all predictors across both outcomes are presented in Table 2, and ROC curves are displayed in Figure 1. For mortality, AIMS-65 demonstrated the highest AUC among all evaluated predictors (0.799, 95% CI 0.696–0.902), followed by ABC (0.731, 95% CI 0.610–0.852) and BUN/Albumin (0.711, 95% CI 0.562–0.859). GBS (0.704), BUN/Hb (0.685), and Rockall (0.611) showed progressively lower AUCs. At its Youden-derived cutoff of ≥6.20, BUN/Albumin achieved 70.6% sensitivity and 82.1% specificity, similar to those of ABC (70.6% and 81.0%, respectively).

For prolonged hospitalization, BUN/Hb showed the numerically highest AUC among all evaluated predictors (0.706, 95% CI 0.660–0.752), followed closely by Rockall (0.695, 95% CI 0.649–0.741) and GBS (0.691, 95% CI 0.645–0.738). ABC (0.674), AIMS-65 (0.666), and BUN/Albumin (0.664) showed modestly lower AUCs. At its optimal cutoff of ≥1.89, BUN/Hb achieved 55.1% sensitivity and 75.3% specificity.

### 3.4. DeLong Pairwise AUC Comparisons

Pairwise AUC comparisons using the DeLong method are presented in Table 3. For mortality, AIMS-65 significantly outperformed BUN/Hb (*p* = 0.038), with AIMS-65 holding the higher AUC. BUN/Albumin showed no statistically significant difference from AIMS-65 (*p* = 0.106), ABC (*p* = 0.374), or GBS (*p* = 0.941), while significantly outperforming Rockall (*p* = 0.024).

For prolonged hospitalization, no statistically significant pairwise differences were detected between both BUN-Based Ratios and any of the four established scores (all *p* > 0.13). BUN/Hb showed numerically higher AUC than all established scores for this outcome, though none of these differences reached statistical significance.

### 3.5. Logistic Regression Analyses

Univariate and multivariate logistic regression results are presented in Table 4. In univariate analysis, both BUN/Hb and BUN/Albumin were significantly associated with both outcomes (all *p* ≤ 0.010). For mortality, BUN/Hb showed an OR of 1.26 per unit (95% CI 1.06–1.51, *p* = 0.010) and BUN/Albumin an OR of 1.18 per unit (95% CI 1.07–1.30, *p* < 0.001). For prolonged hospitalization, BUN/Hb showed an OR of 1.66 per unit (95% CI 1.42–1.93, *p* < 0.001) and BUN/Albumin an OR of 1.22 per unit (95% CI 1.14–1.30, *p* < 0.001). Among established scores, AIMS-65 was most strongly associated with mortality (OR 2.95 per point, *p* < 0.001), while all four scores were significantly associated with prolonged hospitalization. Collinearity assessment demonstrated substantial overlap among BUN, BUN/Hb, BUN/Albumin, and some established scores; however, after excluding overlapping variables from the same models, variance inflation factors in the final multivariable models were within acceptable limits.

In multivariate analysis for mortality, cerebrovascular disease, systolic blood pressure, and CRP were retained as independent predictors in Model A (all *p* < 0.05). Given the limited number of events (*n* = 17), model complexity was deliberately restricted to minimize overfitting, and confidence intervals should be interpreted with caution. In Model B, BUN/Albumin was independently associated with mortality (OR 1.197, 95% CI 1.063–1.347, *p* = 0.003) alongside the same clinical predictors.

For prolonged hospitalization, multivariate analysis identified hemoglobin (OR 0.691 per g/dL, 95% CI 0.628–0.760, *p* < 0.001), INR (OR 3.682 per unit, 95% CI 1.778–7.625, *p* < 0.001), and CRP (OR 1.153 per 10 mg/L, 95% CI 1.048–1.268, *p* = 0.003) as independent predictors. BUN/Hb showed a trend toward significance when added to the multivariate model (OR 1.165, 95% CI 0.981–1.384, *p* = 0.082) but did not meet the prespecified retention threshold of *p* < 0.05.

### 3.6. Model Calibration

Hosmer–Lemeshow calibration test results are presented in Table 5. The primary mortality model demonstrated adequate calibration (Model A: HL χ^2^ = 13.480, df = 8, *p* = 0.096). Mortality Model B showed borderline calibration (*p* = 0.047), likely reflecting limited statistical power at 17 events rather than true model misspecification, given the retained significance of the BUN/Albumin coefficient. For prolonged hospitalization, Model A showed borderline calibration (*p* = 0.038); addition of BUN/Hb improved calibration to *p* = 0.083, suggesting a possible model-level contribution of this ratio despite its non-significant multivariate coefficient.

## 4. Discussion

In this retrospective cohort of patients with acute upper gastrointestinal bleeding, we compared two simple BUN-based ratios, BUN/Hb and BUN/Albumin, with established risk scores for the prediction of in-hospital mortality and prolonged hospitalization. Three main findings emerged. First, AIMS-65 showed the highest discriminative ability for in-hospital mortality. Second, BUN/Albumin remained associated with in-hospital mortality in an exploratory multivariable model, although this finding should be interpreted cautiously because of the limited number of mortality events. Third, BUN/Hb showed the numerically highest AUC for prolonged hospitalization, although this advantage was not statistically significant and did not persist after adjustment. Taken together, these findings suggest that BUN-based ratios may provide useful additional information at presentation, but they do not replace validated scoring systems.

Our mortality results are broadly in line with previous studies showing that score performance in UGIB depends on the endpoint being examined. Recent reviews have emphasized that GBS is particularly useful for identifying low-risk patients and predicting the need for intervention, whereas mortality is often better predicted by scores that capture overall physiological derangement and comorbidity burden [10,14]. In that context, it is not surprising that AIMS-65 yielded the highest AUC for mortality in our cohort. Earlier work has likewise shown that AIMS-65 may outperform GBS for inpatient mortality, even when GBS remains more informative for transfusion-related or intervention-related outcomes [14,15]. Our findings therefore support the idea that mortality in UGIB is not determined by bleeding severity alone, but also by the patient’s general physiological reserve.

The role of the ABC score also deserves consideration. In the international derivation and validation study, ABC was developed specifically to improve mortality prediction in gastrointestinal bleeding and showed strong performance for that endpoint [6,16]. In our cohort, ABC also performed well, although AIMS-65 had the highest AUC numerically. Rather than viewing this as a contradiction, it is more reasonable to interpret it as cohort-dependent variation. Differences in age distribution, comorbidity burden, disease severity, admission practices, and endpoint definition can all influence score performance across studies. In practical terms, our data suggest that AIMS-65 and ABC both remain clinically relevant for mortality assessment, while the BUN-based ratios should be considered supportive markers rather than clear alternatives to these established tools.

One of the more interesting findings in our study was the association between BUN/Albumin and in-hospital mortality. This is consistent with prior work on the blood urea nitrogen-to-albumin ratio in gastrointestinal bleeding [17]. Bae et al. reported that BUN/Albumin was a simple and useful predictor in elderly patients with gastrointestinal bleeding, with performance comparable to AIMS-65 for adverse outcomes [18]. Other studies have also shown that the BUN/Albumin ratio is associated with ICU requirement and in-hospital mortality, again suggesting that it captures clinically meaningful risk beyond bleeding alone [8]. Our results fit well with that literature. In the present study, BUN/Albumin showed moderate discrimination for mortality and did not statistically outperform AIMS-65, ABC, or GBS. Therefore, its prognostic role should be interpreted as supportive and exploratory rather than equivalent or superior to established risk scores. This supports the view that the ratio may reflect a broader vulnerability profile in UGIB.

That interpretation is biologically plausible. BUN elevation in UGIB has traditionally been linked to upper gastrointestinal blood digestion and absorption, intravascular volume depletion, and reduced renal perfusion. When BUN is interpreted together with serum albumin, the resulting ratio may also incorporate information related to nutritional status, systemic inflammation, frailty, and reduced physiological reserve [19]. This may explain why BUN/Albumin appeared to be more closely related to mortality than BUN/Hb in our cohort. Importantly, BUN/Albumin did not statistically outperform AIMS-65, ABC, or GBS for mortality prediction; therefore, its value should be interpreted as complementary rather than competitive. The pairwise AUC comparisons did not show significant differences versus AIMS-65, ABC, or GBS, and the low number of deaths requires a cautious reading of all mortality-related analyses.

The findings for prolonged hospitalization followed a somewhat different pattern. BUN/Hb showed the numerically highest AUC for this outcome, although the difference from the established scores was not statistically significant and the ratio did not remain independently associated after multivariable adjustment. Because both BUN and hemoglobin are components of the GBS, the prognostic information provided by BUN/Hb may partly overlap with that of GBS. This overlap should be considered when interpreting the apparent performance of BUN/Hb. For this reason, the present findings should not be interpreted as demonstrating superiority over established risk scores. Rather, these ratios may be viewed as simple complementary markers that can be calculated immediately from routine laboratory tests at presentation and may provide additional information during the initial assessment of patients. Even so, the signal is clinically understandable. A ratio combining BUN and hemoglobin may capture the immediate burden of bleeding more directly than BUN/Albumin by integrating a marker related to upper GI blood digestion and hypovolemia with a marker of anemia severity at presentation. Patients with a higher BUN/Hb ratio may therefore be more likely to require transfusion, prolonged stabilization, or closer in-hospital monitoring [20]. At present, however, the literature specifically evaluating BUN/Hb in UGIB appears limited. For that reason, this part of our analysis is best interpreted as exploratory. It may indicate a potentially useful signal, but it does not yet justify firm clinical conclusions.

An additional point worth noting is that the two BUN-Based Ratios appeared to behave differently across outcomes. BUN/Albumin was more closely linked to mortality [8,17,18], whereas BUN/Hb was more closely linked to prolonged hospitalization [2,11]. This pattern may indicate that the two ratios reflect different aspects of clinical severity. BUN/Albumin may be more sensitive to overall systemic vulnerability, while BUN/Hb may better reflect the immediate physiological consequences of bleeding. If this distinction is confirmed in future studies, it could be clinically useful, particularly in the early assessment of patients before full score calculation or endoscopic evaluation.

From a practical perspective, the main strength of these ratios is their simplicity. Both can be calculated from routine admission laboratory tests and do not require endoscopic findings or more complex clinical variables. In busy emergency settings, this may make them attractive as bedside adjuncts. Still, simplicity should not be confused with superiority. Similarly, the Youden-derived cutoff values reported in this study should be considered exploratory and dataset-specific. They should not be used as standalone clinical decision thresholds without prospective external validation, particularly for mortality prediction given the low number of events and limited positive predictive values. Although these ratios are easy to calculate, the present findings do not show that they materially improve current UGIB risk stratification. Therefore, BUN/Hb and BUN/Albumin should be regarded as exploratory supportive markers rather than tools that should change current clinical practice. In our cohort, the established scores remained at least comparable overall and AIMS-65 performed best for mortality. The most appropriate conclusion, therefore, is that BUN/Hb and BUN/Albumin may supplement early risk assessment rather than replace validated tools. BUN/Albumin and BUN/Hb showed modest discriminatory ability and may represent exploratory bedside markers. However, their incremental value over validated risk scores remains unproven and requires external validation.

Several limitations should be acknowledged. This was a retrospective single-center study, which may limit generalizability. The number of in-hospital deaths was low, reducing statistical power for mortality analyses and increasing uncertainty around multivariable estimates. Although endoscopic findings were collected and reported, Forrest classification and high-risk stigmata were not incorporated as separate variables in the final multivariable models. This should be considered a limitation, as endoscopic severity may influence prognosis, rebleeding risk, transfusion requirement, and hospital resource use. Therefore, the observed association between BUN/Albumin and in-hospital mortality should be interpreted as exploratory and hypothesis-generating rather than as definitive evidence of independent prognostic value. In addition, several clinically relevant UGIB outcomes, including transfusion requirement, need for endoscopic therapy, ICU admission, rebleeding, and composite adverse outcomes, were not consistently available in the retrospective dataset and therefore could not be analyzed. Future prospective studies should evaluate the performance of BUN-based ratios for these outcomes. Another limitation of the present study is that prolonged hospitalization was used as a study outcome. Although prolonged hospitalization was selected as a clinically relevant outcome, length of stay is influenced by multiple factors and does not solely reflect the severity of bleeding. In addition to the clinical course of upper gastrointestinal bleeding, comorbid conditions, transfusion requirements, discharge planning, local discharge practices, institutional workflow, institutional practices, and healthcare resource availability may all contribute to longer hospitalization. Therefore, prolonged hospitalization should be interpreted as a multifactorial outcome rather than a direct measure of bleeding severity. In addition, model calibration was not uniformly optimal across all analyses, which supports a cautious interpretation. Finally, although BUN/Hb showed a potentially interesting signal for prolonged hospitalization, direct comparative literature for this ratio in UGIB remains limited and external validation is needed.

## 5. Conclusions

In conclusion, our findings suggest that simple BUN-based ratios may offer additional bedside prognostic information in acute UGIB. BUN/Albumin was associated with in-hospital mortality and showed modest discriminatory ability, but it did not demonstrate statistically significant superiority over most established scores. BUN/Hb showed the numerically highest discrimination for prolonged hospitalization; however, this finding should not be interpreted as evidence of superiority because pairwise AUC differences were not statistically significant and the association did not persist in multivariable analysis. Given the limited number of mortality events, these findings require confirmation in larger prospective studies before firm conclusions can be drawn. Overall, these ratios appear to have potential as practical complementary markers, but established scoring systems remain central to UGIB risk stratification. Importantly, the present analyses were not designed to prove formal incremental prognostic value beyond validated risk scores; therefore, BUN/Hb and BUN/Albumin should be regarded as exploratory adjunctive markers rather than replacements for GBS, AIMS-65, ABC, or Rockall scores. However, the present study does not demonstrate that these ratios materially improve current risk stratification or support changes in clinical practice at this stage. Although the present findings are encouraging, external validation in independent multicenter cohorts is required before these markers can be incorporated into routine clinical decision-making.

## Figures and Tables

**Table 1:** Baseline demographic, clinical, laboratory, and endoscopic characteristics of the study population (*n* = 486).

Variable	Median (IQR)	Mean ± SD/Range
General Characteristics		
Total patients, *n*	486	
Age (years)	67 (49–79)	63.7 ± 19.0/21–95
Male sex, *n* (%)	292 (60.1%)	
Length of hospital stay (days)	5 (2–10)	7.1 ± 8.1/0–77
Age Groups		
<40 years, *n* (%)	75 (15.4%)	
40–60 years, *n* (%)	115 (23.7%)	
60–75 years, *n* (%)	139 (28.6%)	
>75 years, *n* (%)	157 (32.3%)	
Comorbidities		
Hypertension, *n* (%)	238 (49.0%)	
Coronary artery disease, *n* (%)	149 (30.7%)	
Diabetes mellitus, *n* (%)	133 (27.4%)	
Atrial fibrillation, *n* (%)	59 (12.1%)	
History of cerebrovascular disease, *n* (%)	26 (5.3%)	
Dementia, *n* (%)	14 (2.9%)	
Medication Use		
Antihypertensive agents, *n* (%)	224 (46.1%)	
Antiplatelet agents, *n* (%)	158 (32.5%)	
Anticoagulants, *n* (%)	111 (22.8%)	
Oral antidiabetic agents, *n* (%)	100 (20.6%)	
Statins, *n* (%)	59 (12.1%)	
Insulin, *n* (%)	19 (3.9%)	
Presenting Symptoms		
Melena, *n* (%)	353 (72.6%)	
Hematemesis, *n* (%)	196 (40.3%)	
Syncope, *n* (%)	52 (10.7%)	
Hematochezia, *n* (%)	47 (9.7%)	
Vital Signs		
Heart rate (beats/min)	86 (76–97)	87.9 ± 16.9/54–175
Systolic blood pressure (mmHg)	123 (112–134)	123.2 ± 20.6/60–208
Diastolic blood pressure (mmHg)	70 (60–76)	69.0 ± 12.0/33–100
Hematological Parameters		
Admission hemoglobin (g/dL)	8.80 (7.20–11.00)	9.1 ± 2.7/3.6–15.7
White blood cell count (×10^3^/µL)	9.19 (7.14–11.67)	9.6 ± 3.9/2.2–35.6
Neutrophil count (×10^3^/µL)	6.76 (4.89–9.11)	7.2 ± 3.6/1.2–30.8
Lymphocyte count (×10^3^/µL)	1.52 (1.08–2.17)	1.7 ± 0.9/0.1–6.8
Monocyte count (×10^3^/µL)	0.46 (0.35–0.60)	0.51 ± 0.37/0.06–5.40
Platelet count (×10^3^/µL)	265.5 (208–317)	272.7 ± 100.3/58–680
Red cell distribution width (%) *	14.9 (13.8–16.9)	—
Neutrophil-to-lymphocyte ratio	4.05 (2.62–7.22)	5.7 ± 5.0/0.8–40.6
Platelet-to-lymphocyte ratio	172.1 (119.1–234.6)	206.5 ± 156.7/41.3–1971
Lymphocyte-to-monocyte ratio	3.36 (2.37–4.71)	3.8 ± 2.5/0.1–31.8
Monocyte-to-lymphocyte ratio	0.30 (0.21–0.42)	0.39 ± 0.48/0.03–8.29
Biochemical Parameters		
BUN (mg/dL)	13.27 (8.97–20.02)	16.0 ± 10.3/2.2–79.6
Creatinine (mg/dL)	0.93 (0.74–1.15)	1.02 ± 0.43/0.46–4.50
Albumin (g/dL)	3.80 (3.40–4.10)	3.78 ± 0.53/2.40–5.30
INR	1.10 (1.00–1.20)	1.36 ± 1.24/0.80–10.90
C-reactive protein (mg/L)	5.0 (2.0–14.0)	17.2 ± 37.0/0.5–350
AST (U/L)	19 (13–28)	49.9 ± 296.8/4–3787
ALT (U/L)	19 (14–28)	38.0 ± 163.5/1–2085
ALP (U/L)	64 (53–85)	77.0 ± 52.5/19–450
GGT (U/L)	21 (15–34)	37.4 ± 64.0/5–943
LDH (U/L)	227 (190–303)	300.9 ± 491.9/94–5793
Total bilirubin (mg/dL)	0.50 (0.30–0.70)	0.63 ± 0.55/0.10–6.20
Direct bilirubin (mg/dL)	0.10 (0.10–0.20)	0.21 ± 0.24/0.10–2.60
Established Scoring Systems		
GBS	7 (2–9)	6.3 ± 4.2/0–18
AIMS-65 Score	1 (0–1)	0.83 ± 0.83/0–4
ABC Score	2 (1–4)	2.6 ± 2.0/0–9
Rockall Score	4 (2–5)	3.6 ± 1.9/0–9
BUN-Based Ratios		
BUN/Hb ratio	1.62 (0.92–2.54)	2.02 ± 1.70/0.25–14.38
BUN/Albumin ratio	3.68 (2.29–5.61)	4.47 ± 3.24/0.57–21.52
Endoscopic Findings		
Peptic ulcer (Forrest 1A–3), *n* (%)	247 (50.8%)	
Active arterial bleeding—Forrest 1A, *n* (%)	4 (0.8%)	
Oozing hemorrhage—Forrest 1B, *n* (%)	34 (7.0%)	
Non-bleeding visible vessel—Forrest 2A, *n* (%)	34 (7.0%)	
Adherent clot—Forrest 2B, *n* (%)	21 (4.3%)	
Hematin-covered base—Forrest 2C, *n* (%)	30 (6.2%)	
Clean-based ulcer—Forrest 3, *n* (%)	124 (25.5%)	
Gastritis, *n* (%)	189 (38.9%)	
Esophagitis, *n* (%)	23 (4.7%)	
Normal endoscopy, *n* (%)	21 (4.3%)	
Angiodysplasia, *n* (%)	5 (1.0%)	
Mallory-Weiss tear, *n* (%)	1 (0.2%)	
High-risk stigmata (Forrest 1A–2B), *n* (%)	93 (19.1%)	
Low-risk stigmata (Forrest 2C–3), *n* (%)	154 (31.7%)	
Clinical Outcomes		
In-hospital mortality, *n* (%)	17 (3.5%)	
Prolonged hospitalization (≥7 days), *n* (%)	207 (42.6%)	

Notes: IQR: interquartile range; SD: standard deviation; BUN: blood urea nitrogen; Hb: hemoglobin; INR: international normalized ratio; AST: aspartate aminotransferase; ALT: alanine aminotransferase; ALP: alkaline phosphatase; GGT: gamma-glutamyl transferase; LDH: lactate dehydrogenase; GBS: Glasgow–Blatchford score; AIMS-65: albumin, international normalized ratio, mental status, systolic blood pressure, and age ≥ 65 years; ABC: age, blood tests, and comorbidities score. All continuous variables showed non-normal distributions according to the Shapiro–Wilk test; therefore, median and interquartile range were used as the primary descriptive measures. * Two implausible RDW values were treated as missing. BUN values were calculated from serum urea values using the standard conversion formula: BUN (mg/dL) = serum urea (mg/dL) × 0.467.

**Table 2:** Diagnostic performance of BUN-based ratios and established scoring systems for predicting clinical outcomes.

Predictor	AUC (95% CI)	Cutoff	Sensitivity	Specificity	PPV	NPV
Mortality (*n* = 486; Events = 17)						
GBS	0.704 (0.593–0.815)	≥7	94.1%	47.3%	6.1%	99.6%
AIMS-65	0.799 (0.696–0.902)	≥2	64.7%	84.4%	13.1%	98.5%
ABC	0.731 (0.610–0.852)	≥5	70.6%	81.0%	11.9%	98.7%
Rockall	0.611 (0.459–0.764)	≥8	23.5%	97.4%	25.0%	97.2%
BUN/Hb	0.685 (0.567–0.803)	≥2.58	64.7%	78.0%	9.6%	98.4%
BUN/Albumin	0.711 (0.562–0.859)	≥6.20	70.6%	82.1%	12.5%	98.7%
Prolonged hospitalization ≥ 7 days (*n* = 486; events = 207)						
GBS	0.691 (0.645–0.738)	≥5	79.7%	46.6%	52.5%	75.6%
AIMS-65	0.666 (0.621–0.711)	≥1	75.8%	49.8%	52.9%	73.5%
ABC	0.674 (0.626–0.722)	≥4	45.9%	81.0%	64.2%	66.9%
Rockall	0.695 (0.649–0.741)	≥3	87.0%	40.9%	52.2%	80.9%
BUN/Hb	0.706 (0.660–0.752)	≥1.89	55.1%	75.3%	62.3%	69.3%
BUN/Albumin	0.664 (0.616–0.713)	≥3.46	69.6%	60.2%	56.5%	72.7%

Notes: AUC: area under the ROC curve; CI: confidence interval; PPV: positive predictive value; NPV: negative predictive value; GBS: Glasgow–Blatchford Score; AIMS-65: Albumin, Mental status, Systolic blood pressure, age ≥ 65; ABC: Acuity of Upper GI Bleeding score; BUN: blood urea nitrogen (mg/dL); Hb: admission hemoglobin. Cutoff values determined by the Youden index (J = sensitivity + specificity − 1). 95% CIs computed by the DeLong method.

**Table 3:** DeLong pairwise comparisons of AUCs: BUN-based ratios versus established scoring systems.

Outcome	BUN-Based Ratio	Comparator Score	Higher AUC	*p*-Value
Mortality	BUN/Hb	GBS	BUN/Hb	0.148
Mortality	BUN/Hb	AIMS-65	AIMS-65	0.038 *
Mortality	BUN/Hb	ABC	ABC	0.116
Mortality	BUN/Hb	Rockall	BUN/Hb	0.794
Mortality	BUN/Albumin	GBS	BUN/Albumin	0.941
Mortality	BUN/Albumin	AIMS-65	AIMS-65	0.106
Mortality	BUN/Albumin	ABC	ABC	0.374
Mortality	BUN/Albumin	Rockall	BUN/Albumin	0.024 *
Prolonged hospitalization	BUN/Hb	GBS	BUN/Hb	0.656
Prolonged hospitalization	BUN/Hb	AIMS-65	BUN/Hb	0.132
Prolonged hospitalization	BUN/Hb	ABC	BUN/Hb	0.130
Prolonged hospitalization	BUN/Hb	Rockall	BUN/Hb	0.464
Prolonged hospitalization	BUN/Albumin	GBS	GBS	0.266
Prolonged hospitalization	BUN/Albumin	AIMS-65	AIMS-65	0.938
Prolonged hospitalization	BUN/Albumin	ABC	ABC	0.633
Prolonged hospitalization	BUN/Albumin	Rockall	Rockall	0.227

Notes: *p*-values derived from DeLong pairwise comparison of AUCs. ‘Higher AUC’ indicates which predictor had the numerically higher AUC in each comparison. GBS: Glasgow–Blatchford Score; AIMS-65: Albumin, Mental status, Systolic blood pressure, age ≥ 65; ABC: Acuity of Upper GI Bleeding score; BUN: blood urea nitrogen; Hb: admission hemoglobin. * *p* < 0.05.

**Table 4:** Univariate and multivariate logistic regression analyses for in-hospital mortality and prolonged hospitalization.

Variable	Univariate OR (95% CI)	*p*	Multivariate OR (95% CI)	*p*
	Model A/Model B †			
Mortality (*n* = 486; Events = 17) ‡				
Age (per 5 years)	1.15 (0.99–1.34)	0.067	—	—
Cerebrovascular disease	8.89 (2.87–27.6)	<0.001	5.28 (1.20–23.2)/6.81 (1.57–29.5)	0.027/0.010
Systolic BP (per 10 mmHg)	0.55 (0.43–0.69)	<0.001	0.628 (0.476–0.829)/0.695 (0.529–0.913)	0.001/0.009
Hemoglobin (per 1 g/dL)	0.74 (0.59–0.92)	0.007	—	—
BUN (per 10 mg/dL)	1.43 (1.03–1.99)	0.034	—	—
Creatinine (per 0.5 mg/dL)	1.71 (1.20–2.43)	0.003	—	—
Albumin (per 0.5 g/dL)	0.18 (0.10–0.33)	<0.001	—	—
Platelet (per 50 × 10^3^/µL)	0.68 (0.49–0.94)	0.018	—	—
WBC (per 2 × 10^3^/µL)	1.34 (1.13–1.58)	<0.001	—	—
NLR (per 5 units)	1.77 (1.31–2.37)	<0.001	—	—
CRP (per 10 mg/L)	1.20 (1.10–1.29)	<0.001	1.178 (1.088–1.275)/1.211 (1.115–1.316)	<0.001/<0.001
AIMS-65 score (per 1 point)	2.95 (1.84–4.73)	<0.001	—	—
ABC score (per 1 point)	1.53 (1.20–1.96)	<0.001	—	—
GBS (per 1 point)	1.19 (1.06–1.34)	0.004	—	—
BUN/Hb ratio (per 1 unit)	1.26 (1.06–1.51)	0.010	—	—
BUN/Albumin ratio (per 1 unit) †	1.18 (1.07–1.30)	<0.001	—/1.197 (1.063–1.347)	—/0.003
Prolonged hospitalization ≥ 7 days (*n* = 486; events = 207)				
Age (per 5 years)	1.15 (1.10–1.21)	<0.001	—	—
Diabetes mellitus	1.75 (1.17–2.62)	0.006	—	—
Coronary artery disease	2.08 (1.40–3.07)	<0.001	—	—
Atrial fibrillation	3.01 (1.70–5.33)	<0.001	—	—
Anticoagulant use	4.04 (2.57–6.36)	<0.001	—	—
Heart rate (per 10 bpm)	1.18 (1.06–1.32)	0.003	—	—
Hemoglobin (per 1 g/dL)	0.67 (0.61–0.73)	<0.001	0.691 (0.628–0.760)	<0.001
BUN (per 10 mg/dL)	1.71 (1.39–2.11)	<0.001	—	—
Creatinine (per 0.5 mg/dL)	1.62 (1.27–2.06)	<0.001	—	—
Albumin (per 0.5 g/dL)	0.47 (0.38–0.58)	<0.001	—	—
INR (per 1 unit)	7.95 (3.23–19.6)	<0.001	3.682 (1.778–7.625)	<0.001
NLR (per 5 units)	1.26 (1.05–1.52)	0.013	—	—
CRP (per 10 mg/L)	1.17 (1.08–1.27)	<0.001	1.153 (1.048–1.268)	0.003
GBS (per 1 point)	1.18 (1.13–1.24)	<0.001	—	—
AIMS-65 score (per 1 point)	2.35 (1.83–3.03)	<0.001	—	—
ABC score (per 1 point)	1.38 (1.25–1.52)	<0.001	—	—
Rockall score (per 1 point)	1.52 (1.36–1.69)	<0.001	—	—
BUN/Hb ratio (per 1 unit)	1.66 (1.42–1.93)	<0.001	— §	—
BUN/Albumin ratio (per 1 unit)	1.22 (1.14–1.30)	<0.001	—	—

Notes: OR: odds ratio; CI: confidence interval; Hb: admission hemoglobin; BUN: blood urea nitrogen (mg/dL); WBC: white blood cell count; NLR: neutrophil-to-lymphocyte ratio; CRP: C-reactive protein; BP: blood pressure; GBS: Glasgow–Blatchford Score; AIMS-65: Albumin, INR, Mental status, Systolic blood pressure, age ≥ 65; ABC: Acuity of Upper GI Bleeding score; INR: international normalized ratio. Continuous variables are reported per clinically meaningful unit increments. Univariate analysis was performed for all candidate variables; final multivariate models were constructed by backward stepwise selection (retention threshold *p* < 0.05). † For mortality: Model A includes cerebrovascular disease, systolic BP, and CRP; Model B adds BUN/Albumin ratio. Results are shown as Model A/Model B. ‡ Mortality models should be interpreted with caution given the limited event count (*n* = 17); wide confidence intervals reflect inherent model instability. § BUN/Hb ratio was significant in univariate analysis for prolonged hospitalization (OR 1.66, 95% CI 1.42–1.93, *p* < 0.001) but did not retain significance in the multivariate model (OR 1.165, 95% CI 0.981–1.384, *p* = 0.082).

**Table 5:** Hosmer–Lemeshow goodness-of-fit test results for logistic regression models.

Model	*n*	Events	HL χ^2^	df	*p*-Value	Calibration
Mortality						
Model A: Cerebrovascular disease + Systolic BP + CRP	486	17	13.480	8	0.096	Adequate
Model B: Model A + BUN/Albumin ratio	486	17	15.692	8	0.047	Borderline ‡
Prolonged hospitalization (≥7 days)						
Model A: Hemoglobin + INR + CRP	486	207	16.284	8	0.038	Borderline §
Model B: Model A + BUN/Hb ratio	486	207	13.971	8	0.083	Adequate

Notes: HL: Hosmer–Lemeshow; df: degrees of freedom; BP: blood pressure; BUN: blood urea nitrogen; Hb: admission hemoglobin; INR: international normalized ratio; CRP: C-reactive protein. The Hosmer–Lemeshow test compares observed vs. expected event rates across 10 probability deciles; *p* > 0.05 indicates adequate calibration. ‡ Mortality Model B showed borderline calibration (*p* = 0.047), most likely attributable to the small event count (*n* = 17) rather than true model misspecification, given the retained significance of the BUN/Albumin coefficient. § Prolonged hospitalization Model A showed borderline calibration (*p* = 0.038); addition of BUN/Hb in Model B improved calibration to *p* = 0.083, suggesting a possible model-level contribution of this ratio despite its non-significant multivariate coefficient (*p* = 0.082).

## Data Availability

The data underlying this article will be shared upon reasonable request to the corresponding author.
